# Mechanical thrombectomy for AIS from large vessel occlusion – current trends and future perspectives

**DOI:** 10.1097/MS9.0000000000001385

**Published:** 2023-10-04

**Authors:** Gauri Parvathy, Rohit C. Dey, Lakshmi Venkata Simhachalam Kutikuppala, Aakansh R. Maheshwari, Elwy Josey, Jyothi S. Chintala, Mohammed Abdullah, Swathi Godugu

**Affiliations:** a Department of Internal Medicine, Tbilisi State Medical University, Tbilisi, Georgia; b Department of Internal Medicine, Altai State Medical University, Barnaul, Russia; c Department of General Surgery, Dr NTR University of Health Sciences, Vijayawada; d Department of Anesthesiology, Dr Pinnamaneni Siddhartha Institute of Medical Sciences and Research Foundation, Chinaoutpalli, Andhra Pradesh; e Department of Internal Medicine, Pacific Medical College and Hospital, Rajasthan, India; f Department of Internal Medicine, Dubai Academic Health Corporation; g Department of Internal Medicine, Saudi German Hospital, Dubai, UAE; h Department of Internal Medicine, Zaporozhye State Medical University, Zaporozhye, Ukraine

**Keywords:** acute ischemic stroke (AIS), large vessel occlusion (LVO), mechanical thrombectomy, stroke

## Abstract

Stroke is found to be one of the global top causes of mortality and the major factor in years of life with a handicap (DALYs). Ischemic strokes contributed to nearly 70% of all strokes worldwide. For endovascular thrombectomy in acute ischemic stroke with large vessel obstruction (AIS-LVO), using stent retrievers and/or reperfusion catheters has become the gold standard of therapy. The methodology involved keyword-based search in databases like PubMed, Embase, and Google Scholar for recent publications on mechanical thrombectomy (MT), AIS, large vessel occlusion (Large Vessel Occlusion (LVO)), screening relevant articles, retrieving full texts, and synthesizing key findings on procedural advancements, patient selection, COVID-19 (coronavirus disease 2019) impact, delay effects, effectiveness, clinical outcomes, and future perspectives. Only people with substantial cerebral artery obstruction may do well from MT. This includes the distal carotid artery and the proximal middle cerebral artery (segment M1). The size of a blocked vessel and NIHSS (National Institute of Health Stroke Scale) score are directly connected. Both the 2018 and 2019 versions of the AHA/ASA (American Heart Association/American Stroke Association) Guidelines for the Early Management of Patients with Acute Ischemic Stroke contained the recommendations that cases with AIS-LVO get endovascular therapy when administered during the time frame of 0–6 h after onset (Grade IA evidence). It is questionable whether this group of patients can be managed without the need for intravenous tissue plasminogen activator at the onset. When functional independence [modified Rankin Scale (mRS) score 2] was present at long-term follow-up, the endovascular intervention was favored. Tenecteplase, which differs from alteplase in terms of genetic variation, has a greater half-life and a higher level of fibrin selectivity, enabling bolus infusion. Studies have also demonstrated its efficacy and safety, as well as its long-term cost-effectiveness.

## Introduction

Highlights

- Stroke continues to be the second leading cause of death worldwide and the major factor in years of life with a handicap (DALYs).
- For acute ischemic stroke-large vessel obstruction (AIS-Large Vessel Occlusion), endovascular thrombectomy (EVT) using stent retrievers and/or reperfusion catheters has emerged as the gold standard of therapy.
- Tenecteplase (TNK), a genetically modified variant of alteplase, has a longer half-life and a higher level of fibrin selectivity, enabling bolus infusion.
- Studies have also demonstrated efficacy and safety, as well as the long-term cost-effectiveness of TNK.
- When compared to standard medical treatment alone, mechanical thrombectomy appears to be effective and safe for the treatment of patients with AIS-Large Vessel Occlusion.

Acute ischemic stroke (AIS) stands as a prominent contributor to both mortality and disability. Acute therapy for individuals who get a rapid diagnosis may include an effort to restore blood flow. Despite being regularly used to accomplish this goal for around 20 years, intravenous thrombolysis (IVT) has two major drawbacks: it must be delivered within 4–5 h of the onset of manifestations, and it has various contraindications (e.g. active bleeding, recent surgery, coagulation abnormalities)^1^. IVT administered within 4–5 h after the start of manifestations is the primary treatment for ischemic stroke^2^. Stroke remains the second leading cause of mortality on a global scale and represents a significant contributor to years lived with disability (DALYs). Ischemic strokes constituted 71% of the total incidence of strokes on a global level. Due to technological developments in innovative thrombectomy devices and interventional therapy, reperfusion catheters and/or endovascular thrombectomy (EVT) with stent retrievers have recently been established as the gold standard of care for AIS with large vessel obstruction (AIS-LVO) of the anterior circulation. According to estimates, Large Vessel Occlusion (LVO) accounts for roughly 30% of AISs in the USA, and 30–40 patients per 100 000 annually have clots in places that qualify for EVT (ICA/M-1, M-2, basilar)^3^.

The conventional therapy for large vessel occlusion (LVO) in AIS is mechanical thrombectomy (MT). SYNTHESIS Expansion, IMS III, and MR RESCUE, the first three significant randomized control studies (RCTs) juxtaposing endovascular therapy to conventional intravenous therapy, found no differences in clinical results between the given treatment modalities. The studies were limited in their methodology due to the absence of specific criteria for selecting major vessel occlusion (LVO) cases and the exclusion of stent retrievers in the endovascular therapy group (13% SYNTHESIS Expansion, 2% IMS III, and 0% MR RESCUE)^4^. The following five randomized clinical trials (‘SWIFT PRIME, MR CLEAN, ESCAPE, REVASCAT, and EXTEND-IA’) addressed these constraints, and the results showed a notable enhancement in clinical outcomes and recanalization rates when compared to the use of medical therapy alone to other interventions^5^. MT has gained widespread acceptance as the prevailing therapeutic strategy for the management of AIS.

## Methodology

The search protocol involves identifying relevant keywords such as mechanical thrombectomy, AIS, large vessel occlusion, and current trends/future perspectives. Databases like PubMed, Embase, and Google Scholar are searched using a combination of keywords and Boolean operators. The search is limited to recent publications. Relevant articles are screened, and their full texts are retrieved for critical appraisal. Key findings regarding procedural advancements, patient selection, COVID-19 (coronavirus disease 2019) patients, the effect of delay, effectiveness, and clinical outcomes are synthesized, along with discussions on future perspectives and emerging developments in the field.

## Selection criteria

Only individuals with an obstruction in a significant cerebral artery may get treated with MT because of its technical nature. This comprises the distal carotid artery and proximal middle cerebral artery (MCA) (segment M1), both of which are blocked in 4–10% of all stroke patients. If it is determined that this is the condition, the case should be sent to a facility with a supra-regional stroke unit that is appropriate. The lack of round-the-clock availability of vascular diagnostics utilizing computed tomography (CT) or magnetic resonance imaging (MRI) angiography could pose challenges. Currently, the prevailing trend leans toward this being a typical occurrence rather than an exceptional one^2^.

The utilization of MT should not be dismissed just based on the advanced age of the patient. Overall, there is not enough support for patient classification based on factors like age, time frame, or NIHSS (National Institute of Health Stroke Scale) score, when taken strictly. In the five published studies on thrombectomy, the mean NIHSS score was 17, indicating that they were individuals with severe impairment. The NIHSS score and the bulk of an occluded artery are clearly correlated. If the NIHSS score is 11 or above, there is a 3.3-fold increased risk of blockage of a major cerebral artery, whereas the NIHSS threshold score is reduced by 1–4 points during the first 3–6 h after stroke. Another study found that an NIHSS score of 8 or above was the most significant determinant of proximal vascular obstruction that was identified, and it exhibited a reduction of 2 units within a 3-h duration^6^.

According to the findings, patients should be sent to a facility for MT within 3 h if their NIHSS score is 9 or higher, and between 3 and 6 h if their score is 7 or higher. This idea was also put out in a consensus paper. Only with agreement between superregional stroke centers and smaller and medium-sized hospitals can such a technique be practical in Germany.

## MT performed within guidelines

The American Heart Association/American Stroke Association Guidelines for the Early Management of Patients With Acute Ischemic Stroke in both 2018 and 2019 incorporated a recommendation (supported by Grade IA evidence) advocating for the administration of endovascular therapy to patients with AIS-LVO who present within the time frame of 0–6 h after the onset of symptoms (Grade IA evidence). MT should be administered to AIS-LVO patients who satisfy all the following requirements: prestroke modified Rankin Scale (mRS) score of 0–1, age of 18, NIHSS score of 6, and causal blockage of the internal carotid artery or MCA segment 1 (M1). The criteria call for an initial CT score (ASPECTS) of 6 and the capacity to initiate treatment (groin puncture) within 6 h of symptom onset^3^. The DEFUSE-3 trial reported a discrepancy characterized by a reduced infarct core size accompanied by an enlarged ischemic penumbra. This finding was supported by a Grade IIa recommendation and Grade B-R evidence, indicating the efficacy of endovascular therapy following imaging evaluation for ischemic stroke. Patients who have experienced AIS-LVO and present within a time window of 6–16 h should get endovascular therapy. In five randomized controlled trials (RCTs) that treated patients with AIS-LVO within 6 h of manifestation onset (‘SWIFT PRIME, MR CLEAN, REVASCAT, ESCAPE, and EXTEND-IA’), as well as in the DEFUSE-3 and DAWN trials that treated patients with AIS-LVO between 6 and 24 h of onset, the stent retriever has emerged as the primary thrombectomy instrument employed in clinical practice^7,8^. According to the guideline update in 2019, it was determined that aspiration as a first-line therapy for AIS-LVO is not inferior to stent-retriever thrombectomy (Grade I recommendation, B-R grade evidence).

Perfusion imaging, as used in the DEFUSE-3 and DAWN trials, has expanded the time interval for MT over the typical 6-h limit. Perfusion imaging evaluates blood flow and identifies salvageable brain tissue in patients with blocked vessels. By assessing the ischemic penumbra and identifying a ‘mismatch’ between the core infarct and surrounding tissue, physicians can determine candidates for MT beyond the standard time frame. Perfusion imaging extends the treatment window, improving patient outcomes by expanding eligibility for the procedure. However, the use of perfusion imaging requires specialized expertise and resources for interpretation, and a multidisciplinary team should be involved in decision-making^9^.

## AIS-LVO in patients with COVID-19

The COVID-19 pandemic has had a tremendous impact on the provision of stroke care on a global scale. Patients who have experienced an acute stroke, either with or without concurrent COVID-19 infection, have necessitated the extension of intervals between screening and subsequent therapy. This is mostly attributed to the silent transmission characteristic of COVID-19. The agreement pertaining to the management and mitigation of COVID-19 among Chinese neurologists underlined the need to optimize the workflow and staffing protocols specifically for acute stroke cases in the context of neurological crises. According to a comprehensive database in Germany, individuals diagnosed with AIS who also contracted COVID-19 exhibited a significantly higher proportion of in-hospital mortality compared to those without COVID-19 (22.5% vs. 7.8%, respectively). Conversely, the rate of MT was shown to be lower in AIS patients with COVID-19 compared to those without (3.8% vs. 7.9%, respectively). Furthermore, the European Multicenter Study of ET-COVID-19 unveiled that individuals diagnosed with AIS who were administered COVID-19 treatment exhibited a notably elevated 30-day mortality rate (29%) after undergoing MT^10^. Given the current trajectory of the pandemic, it is imperative that we expeditiously explore a novel approach to optimizing machine translation (MT) for AIS-LVO in the context of COVID-19.

## Treatment strategies

### Within 6 h of the beginning of LVO, bridging therapy versus direct MT

If AIS-LVO is identified within 6 h after the onset of symptoms, the recommended approach for treatment is the administration of bridging therapy. The debate surrounding whether this patient cohort may be effectively handled without the initial administration of intravenous tissue plasminogen activator (IV tPA) has been a topic of discussion. Over the last 10 years, treatments for ischemic stroke have developed quickly. These include MT, intra-arterial (IA) thrombolysis, and systemic IV tPA^11^. IA treatment is still used even though IV tPA administration is still considered the gold standard^5–11^. There are several therapeutic options available for individuals who are candidates for endovascular intervention. There are existing procedures including balloon angioplasty, IA thrombolysis, stenting, aspiration, and mechanical maceration, and new treatments are always being developed^12^.

## Effectiveness of MT

### Outcomes of endovascular versus medical management (MM)

Significant differences between the two arms were found using the Cochran–Mantel–Haenszel test (*P*=0.0143). For the analysis, the scores of 5 and 6 were pooled; 80 (11.5%), 105 (15%), 127 (18.2%), 112 (16%), 54 (7.7%), and 221 (31.6%) of the 699 patients in the intervention arms with long-term follow-up evaluation had mRS scores of 0, 1, 2, 3, 4, and 5, or 6, respectively. In 512 patients in the control arms who had long-term follow-up assessments available, 31 (6%), 71 (13.9%), 87 (17%), 52 (10.1%), and 200 (39.1%) had mRS scores of 0, 1, 2, 3, 4, and 5, or 6, respectively. Endovascular stroke intervention was preferred when functional independence (mRS score 2) was present at long-term follow-up (OR 1.51; 95% CI 1.08–2.12; *P*=0.02)^4^.

### Outcomes after intervention versus MM stratified by LVO criteria

After IMS III was removed, a sensitivity analysis was carried out, only including individuals who had LVO evidence on their baseline neuroimaging (LVO criteria). Functional independence was shown by MR CLEAN and REVASCAT to support endovascular treatment (OR 1.85; 95% CI 1.31–2.63; *P*=0.0005). Functional independence of the two arms was not different according to IMS III alone, without reference to LVO criteria (OR 1.16; 95% CI 0.83–1.62; *P*=0.38). The groupings did not vary significantly from one another (2=3.62; *P*=0.06; *I* ^2^=72.4%)^5^.

### Alteplase or tenecteplase with MT

The two primary thrombolytic medications used to treat AIS patients at this time are alteplase and tenecteplase. A genetically engineered version of alteplase called tenecteplase (TNK) has a longer half-life and a higher degree of fibrin specificity, allowing for bolus infusion. A greater incidence of reperfusion >50% was seen in the Extend-IA TNK trial when IV-TNK was administered instead of IV-alteplase prior to arteriography for MT (22% vs. 10%). Within 4.5 h after the beginning of symptoms, individuals with AIS-LVO treated with TNK had a better independent functional result than those treated with alteplase (‘median mRS, 2 vs. 3, *P*=0.04’)^13^.

### Shortening onset to puncture time may improve outcome

#### Direct transfer to Angio suite

EVT is extremely time-dependent, increasing with each hour of delayed reperfusion reducing the likelihood of 90 days of functional independence by 20%^14^. The first objective of an endovascular procedure for AIS-LVO patients is to shorten the time between the time of onset and the puncture. According to earlier case-controlled research and retrospective observational studies, the median door-to-groin time and the onset-to-groin timings were significantly reduced if a patient with AIS-LVO was taken straight to an Angio suite^15^. Better results were obtained recently, but only with a 13-min faster onset to puncture.

### Combination of MT and IVT in comparison to thrombolysis

For the patients having acute major vascular occlusion (LVO) who are eligible for both IVT and MT, IVT with tPA is the first-line therapy, after which MT is performed. According to the recently released randomized DIRECT-MT trial (‘Direct Intra-Arterial Thrombectomy to Revascularize AIS Patients with Large Vessel Occlusion Efficiently in Chinese Tertiary Hospitals’), the functional outcomes of acute LVO patients did not differ significantly between MT alone and MT preceded by IVT^13^. Even though MT alone appears to be connected with a shorter in-hospital delay prior to the procedure, lower risks of symptomatic intracranial hemorrhage and embolization, and comparable effectiveness to combined IVT+MT for acute LVO, a matched-control study carried out in a real-world practice setting largely supported the findings of the DIRECT-MT trial^16^.

## MT device comparison in terms of effectiveness and safety

### Effectiveness

Widely utilized for this purpose, Solitaire FR (eV3/Covidien Inc., Irvine, California, USA) and Trevo ProVue (Stryker Inc., Kalamazoo, Michigan, USA) have shown great success rates in eliminating clots in major vascular occlusions. The Penumbra System has shown strong effectiveness in clearing clots from smaller veins; however, it could be less successful in clearing blockages from big vessels. Trevo ProVue has shown excellent effectiveness, especially in the removal of big, thick clots^17^.

### Safety

The use of any MT device comes with the possibility of procedural issues such as bleeding, vascular perforation, and distal embolization. The likelihood of problems may differ across devices and rely on several variables, including the patient chosen, the operator’s background, and the treatment carried out. Mechanical aspiration devices have a somewhat greater risk of problems than stent retrievers, according to certain research, albeit this may change based on the patient demographic and treatment^18^.

Both thrombectomy using a stent retriever and aspiration first had comparable final reperfusion rates and functional outcomes. With less usage of rescue devices but a longer time from the groin to reperfusion, stent-retriever thrombectomy was preferable as a stand-alone first-line procedure for achieving reperfusion^17^.

## Long-term outcomes

Due to several variables, including the patient’s age, baseline health state, the severity of the stroke, and other medical problems, the long-term prognosis for individuals treated with MT for AIS might vary greatly^5^. Nevertheless, the following general remarks on the long-term results for individuals who had MT:

- Improved functional outcomes: Patients who successfully undergo MT often have better functional outcomes. This might include increased mobility, the capacity to carry out everyday tasks, and general quality of life.
- Reduced possibility of recurrent stroke: In patients with AIS, MT may help lower the risk of repeat stroke.
- Improved survival: Survival rate may be higher for patients who successfully undergo MT than for those who just get medical care^5^.
- Re-occlusion: Following MT, a clot may sometimes develop again in the treated artery. This may happen in a tiny percentage of people and can need further treatment.

## Effects of delay in MT

The results indicate that MT operation periods exceeding 60 min affect outcomes by increasing complications and device cost rates. These results may help determine whether it would be wise to stop treating a failed thrombectomy case.

A decreased chance of recanalization (TICI 2B or 3), considerably greater device costs, and intraprocedural problems are all linked to prolonged operation timeframes. Importantly, these variations result in worse results at the 90-day follow-up^11^. Patients who had treatments that took more than 60 min had a considerably greater chance of dying or being in a persistent vegetative state, whereas those whose recanalization was completed in less time had a higher chance of having an mRS score of 0–2. In fact, in our experience, each 10-min increase in procedural time had a detrimental effect on the chance of getting a favorable long-term result when clinical considerations were considered^3^. Removing the clot and reestablishing blood flow to the affected area of the brain may become more challenging the longer the stroke is left untreated, decreasing the overall effectiveness of thrombectomy. In such cases of failed recanalization after MT, several treatment options can be considered for rescue efforts. These include IA thrombolytics, rescue stenting, balloon angioplasty, additional MT techniques, and combination therapy^19^.

## Complications of MT

Although MT is emerging as the best method for treating AIS, it has several serious drawbacks. The difficulties are making its therapeutic use more and more difficult. Recent studies show that individuals who undergo MT have a possibility of complications of over 15%^6^. MT has several intraoperative and post-operative issues that must be properly managed in order to optimize the therapeutic benefits of the procedure.

### Primarily intraprocedural complications

Endothelial/nerve damage, infection, and groin hematoma are access-site barriers. Groin hematomas and infections are the most prevalent of these side effects, occurring in 3–12% of patients; neurovascular problems, in contrast, are seen to be rare. Complicacies relating to devices: a major issue that has to be watched out for is vasospasm. It is documented in roughly 4–22% of patients and is caused by the guiding wire irritating the endothelium tissue^18^.

The cervical and cerebral vessels, as well as the puncture site, have a high rate of vascular dissection and perforation. Along with smoking, vessel compliance is a significant contributor to this problem. The significant danger of losing the device, which lengthens the treatment, exists constantly. This lengthening of the process might cause a delay in clot redemption. Subarachnoid hemorrhage, embolization to new or target vascular region, and symptomatic intracerebral hemorrhage (sICH) are further possible complications^20,21^. Extracranial hemorrhage, post-operative bleeding, anesthetic/contrast-related bleeding, and pseudoaneurysm are a few more risks.

### Post-operative complications

Internal challenges that ultimately cause a significant rise in the need for post-operative intensive and stroke treatment. These management hiccups raise costs and postpone the start of recovery. Under the guidance of qualified stroke and neurointervention professionals, these consequences may be reduced and some of them can even be avoided. Although procedural risks from thrombectomy are not insignificant overall, they may be controlled and are within acceptable ranges for emergency treatment^22^.

## Cost-effectiveness

A study found that 267 514 ischemic stroke victims in 32 European nations qualified for MT therapy. MT has been demonstrated to be more effective, cost-effective, and less expensive than conventional treatment in two-thirds of the countries (21/32). In addition to saving $1.7 billion (‘€1.5 billion, 95% uncertainty interval, 1.2–3.6’) in societal costs for Europe, the intervention was predicted to save $981 million (€868 million, 95% uncertainty interval, 1544–2564) in health and social care costs and over 101+327 additional quality-adjusted life years (95% uncertainty interval; 65+180–149+085)^23^.

## Comparison of MT to traditional methods

The strategy used by MT and conventional ischemic stroke treatments to remove a blood clot from a brain blood artery is different. The goal of conventional treatments, such as intravenous thrombolytic drugs, is to dissolve the blood clot or get it to disintegrate naturally. This method is quite straightforward, non-invasive, and generally accessible, although it has the potential to be slow-acting and may not be as successful in clearing big or difficult-to-reach clots. The blood clot is physically removed via a minimally invasive treatment called MT, on the other hand^23^. Large or hard-to-reach clots can usually be removed more successfully using this method, but it is more expensive, time-consuming, and requires specialized medical staff and equipment.

MT has several potential benefits over conventional procedures. The blood clot may be swiftly and efficiently removed, which can greatly enhance results and lessen patient impairment. Second, compared to more intrusive surgical treatments, the process is less invasive and has a low risk of consequences. In contrast to conventional techniques, MT does, however, have significant drawbacks as well. The treatment is more costly in the beginning and necessitates the use of specialized tools and skilled medical workers^25^. Second, it might be difficult to assess the real cost-effectiveness of MT since there are little data on its long-term advantages.

## Future perspectives

The field of MT is one that is rapidly developing and has made significant strides in recent years. As technology and training advance, more healthcare professionals will likely be able to perform the procedure, making it even more widely accessible in the future. The creation of new tools and methods to expedite, secure, and improve the MT procedure is one area of future development. For instance, it is anticipated that robotics and artificial intelligence will be used more frequently in the future, enabling more precise and controlled blood clot removal^26^.

The combination of MT with other therapies, such as endovascular thrombolysis, to offer a comprehensive and efficient treatment approach for ischemic stroke, is another area of future development. In addition to improving the cost-effectiveness of the treatment, this could result in improved patient outcomes and a decrease in disability^25^. The utilization of MT for conditions other than ischemic stroke, like deep vein thrombosis and pulmonary embolism, is also gaining popularity. This offers hope for the creation of fresh, more efficient remedies for these conditions.

## Discussion

A major treatment option for AIS brought on by big artery blockage is MT. The number of research and trials examining the security and efficiency of MT in this patient group has significantly increased in recent years. These trials’ findings, which were generally encouraging, demonstrated that MT, as opposed to conventional therapies such as IVT alone, may improve patient outcomes and minimize disability^2^. Additionally, improvements in patient outcomes and an increase in the frequency of successful mechanical thrombectomies have been brought about by technological and procedural advancements.

Despite the MT’s encouraging outcomes, there are still a few crucial factors to take into mind. To optimize the procedure’s advantages, it should firstly only be carried out by skilled professionals in designated facilities and on schedule. Second, more study is being done to better understand the best techniques for MT, including investigations into the best patient selection, technique, and post-procedure treatment^1^.

Whether individuals with mild ischemic stroke in acute stages brought on by LVO may benefit from thrombectomy is still up for debate. To the best of our knowledge, no RCTs have been conducted on this matter, and observational research has produced contradictory findings. Our findings corroborated the findings of the retrospective research in patients with LVO and moderate symptoms (NIHSS 0–5), thrombectomy was linked to better clinical outcomes than medicinal treatment^27^. Additionally, a 2018 meta-analysis found that thrombectomy had positive results for LVO stroke patients with little or mild symptoms (NIHSS 8)^28^. Nonetheless, the younger age of the patients in the thrombectomy group may have been a significant confounder.

The ETIS registry investigators, on the other hand, found that medical therapy and immediate MT both had equivalent percentages of good functional outcomes at 3 months, with the possibility of delayed MT matched with propensity score, but with about 20% management crossover^29^. Another multicenter cohort research revealed similar findings; however, there was a hint that MT could be helpful for M1 occlusions (*P*=0.07)^28^. According to a recent meta-analysis, individuals with LVO and NIHSS scores below 6 who received the best MM or MT had similar clinical results. Our study contributes to the body of information in this area about the people of eastern China^30^.

Between the MT and MM groups, there was no discernible change in the rate of sICH or death, which is consistent with the results of several matched cohort studies^28^. In a meta-analysis, Goyal *et al*.^30^ found that although sICH rates were comparable between the two groups, MT was linked to increased rates of asymptomatic ICH (OR, 11.07; 95% CI, 1.31–93.53; *P*=0.03). According to Sarraj *et al*., the MT group’s sICH rates were greater and were linked to increased mortality. An elevated risk of sICH may be linked to several MT passes. Since the invention of the thrombectomy procedure about 10 years ago, patients in this research have seen fewer problems and deaths^27^. Overall, primary concerns with MT in patients with mild stroke remain revolve around sICH and associated sequelae.

The encouraging findings of this research might be attributed to several factors. First, the greater recanalization rate in our research (up to 97.7%) was like that reported by Sarraj *et al*. (78%) and Goyal *et al*. in their meta-analysis (84.5%)^28,30^. The BEYOND-SWIFT registry study’s retrospective analysis also suggested that a greater recanalization rate could be linked to a better result in the MT group. Patients whose reperfusion attempts were successful had better results. Second, patients were chosen using perfusion imaging. Regarding the recommendations for thrombectomy in patients with LVO and mild stroke, there were none that were particularly clear in China^30^. The 2015 guideline made no recommendations, while the 2018 guideline allows for cautious consideration of MT following screening and analysis. Patients with smaller infarct cores and greater penumbra may choose thrombectomy even though there are no universal guidelines for choosing a course of therapy for this illness. In addition, factors including family culture and insurance status may influence the choice of therapy. Although we utilized propensity score matching and multivariate logistic regression to account for these factors, indication bias may still have an impact on our findings^3^.

For big vascular occlusion, MT is considered to be the norm. One of the largest and most important investigations was the MR CLEAN study, a multicenter, randomized controlled experiment that included 500 patients with AIS. It contrasted patients who were entitled to regular medical care along with IA therapy with those who did not. The study found that compared to IVT paired with MT, IVT alone had lower functional outcomes and greater disability^32^. These results were validated by further studies, including the ESCAPE trial, which found that MT improved patient outcomes and reduced disability^33^.

In addition to RCTs, meta-analyses and observational studies have also looked at the effectiveness and safety of MT. These studies have generally supported the findings of the randomized trials and shown that MT has a favorable safety profile and a low incidence of procedural issues^5^. AIS patients who had an IA intervention, including MT, experienced a greater rate (13.5%) of functional independence than patients in the control group, demonstrating the safety and effectiveness of this kind of procedure^17^. However, other studies have shown that the danger of intraoperative complications leading to cerebral bleeding is still a concern. Long-term follow-up of the patients who had a history of ischemic stroke revealed additional outcomes, including improvements and positive outcomes in terms of cognitive function and quality of life in terms of health.

Trials that were released especially discuss arterial occlusions proximally with the adjuvants such as ischemic core volumes and collateral circulation, further limiting the individuals who are considered candidates for intervention. To contribute to this expanding body of data, ongoing studies like the ‘SITS Open’, which broadens patient recruitment to include those with posterior circulation occlusions, including occlusions in the basilar artery, are required^34^. The large and unquestionably costly infrastructure needed to support MET is necessary. The technology is still being developed, and the most recent testing was carried out under very idealized conditions (e.g. coordination of teams, time to intervention, etc.). Only if policy, coordination, and resources are scaled effectively will broadening the criteria be beneficial. A more stringent set of criteria (like those used in EXTEND-IA) may be more suitable in a situation like the one described above, where the EXTEND-IA study omitted 22% of eligible participants. owing to physician unavailability^25^.

Additionally, efforts must be made to enhance the standardized software used to find the right patients to give the intervention. Numerous studies have outlined a link between imaging characteristics and the ischemic stroke outcomes. The adoption of RAPID software among the EXTEND-IA and SWIFT PRIME marks the beginning of a standardized eligibility procedure, which will surely result in a rise in its utilization^35^. The patient subgroups classified as wake-up strokes or those beyond the therapy window are neglected in the most current research series. Studies like ‘Perfusion Imaging Selection of Ischemic Stroke Patients for Endovascular Therapy (POSITIVE)’, ‘Trevo and Medical Management versus Medical Management Alone in Wake Up and Late Presenting Strokes (DAWN)’, and ‘Study of Intravenous Thrombolysis with Alteplase in MRI-Selected Patients (MR WITNESS)’, which are ongoing and has the potential to provide important information, will support this hypothesis^36^.

## Conclusion

MT has transformed the treatment of AIS, especially for patients sustaining LVOs. While IVT remains the primary treatment within the first 4.5 h, MT has transformed to be the gold standard for AIS-LVO of the anterior circulation. Studies have shown its effectiveness in improving functional outcomes and increasing reperfusion rates. The use of TNK has demonstrated better independent functional outcomes compared to alteplase. Time is crucial, and shorter onset to puncture times have been related to better outcomes. Direct transfer to an Angio suite has been shown to reduce time delays. However, MT comes with challenges such as access-site barriers and complications. The field of MT continues to advance with the development of new tools and techniques, including robotics and artificial intelligence. The combination of MT with other therapies holds promise for improved patient outcomes and decreased disability. Despite complications, MT has significantly enhanced the management of AIS, and ongoing advancements will further enhance its efficacy and accessibility, ultimately improving the long-term prognosis of the patients.

## Ethical approval

Ethics approval was not required for this review.

## Consent

Informed consent was not required for this review.

## Sources of funding

The authors declare that there has been no funding received for this study and that the authors have not received any financial support or compensation in relation to the preparation and submission of this manuscript.

## Author contribution

G.P. and R.C.D.: study concept, drafting, and editing the paper; L.V.S.K., A.R.M., E.J., J.S.C., and M.A.: drafting and editing the paper; G.S.: drafting, editing, and submitting the paper.

## Conflicts of interest disclosure

The authors declare that there are no conflicts of interest, financial or otherwise, that could influence the results or interpretation of the study. The authors further declare that there has been no financial support or funding received for this study.

## Research registration unique identifying number (UIN)

Not applicable.

## Guarantor

Swathi Godugu.

## Data availability statement

The authors confirm that all data used in the study are available upon request from the corresponding author and can be shared with interested parties. The authors further declare that the data used in the study have been properly documented and stored in accordance with the ethical and legal requirements.

## Provenance and peer review

Not commissioned, externally peer-reviewed.
